# Supplementary material for: Establishment Genes Present on pLS20 Family of Conjugative Plasmids Are Regulated in Two Different Ways
Source: Microorganisms. 2021 Nov 29;9(12):2465. doi: 10.3390/microorganisms9122465 (PMC8708921; doi:10.3390/microorganisms9122465)
Supplement: Supplementary file 1 [file microorganisms-09-02465-s001.zip › microorganisms-1458289-SI.pdf]

Supplemental material

Supplemental Figures

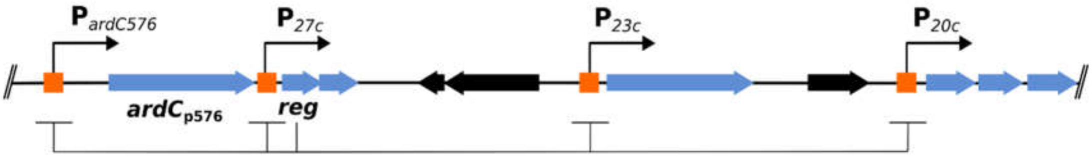

Supplemental Figure S1. Schematic presentation of the the regulatory mechanism of the establishment genes on the pLS20-related plasmid p576. The promoters $P_{ardCp576}$, P_{27c}, P_{23c} and P_{20c} located upstream of four operons on p576 are repressed by the repressor Reg_{p576} (gene indicated as *reg).* Because the repressor protein is not transferred, the promoters will be active after arrival of the DNA into the recipient cell until sufficient Regp576 is produced to repress the promoters again.

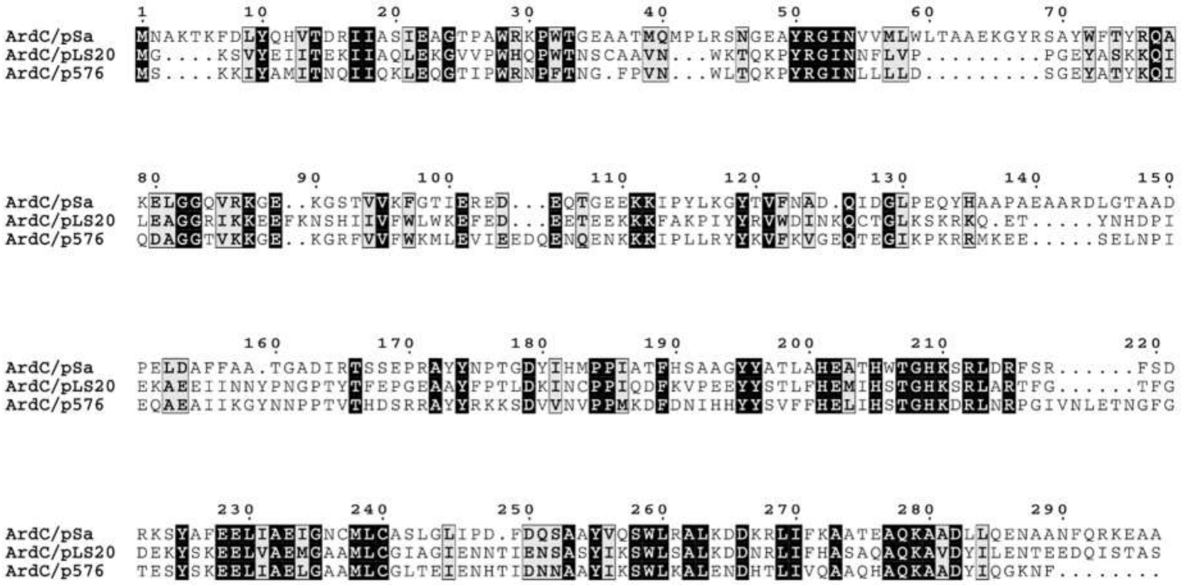

Supplemental Figure S2. Alignment between the *Shigella flexneri* encoded antirestriction protein ArdC (ArdC/pSa) and the putative antirestriction proteins encoded by p576 (ArdC/p576) and pLS20 (ArdC/pLS20). Identical and conserved residues are highlighted in black and grey background, respectively.

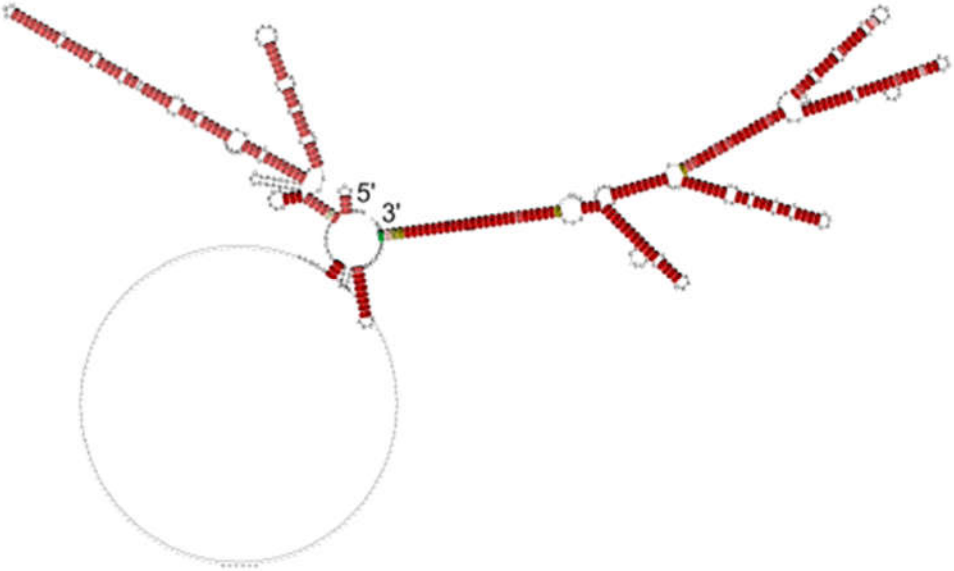

Supplemental Figure S3. Predicted conserved secondary structure of the EGeRS1 sequences when present in ssDNA or RNA. The consensus secondary structure of the five EGeRS1 sequences (see alignment Figure 2B) present on pLS20 was predicted by the RNAalifold weberver and represented as a minimum free energy (MFE) structure. The degree of conservation is indicated using the Vienna RNA conservation colouring scheme. A large ~325 nt branch with four ramifications and located immediately upstream of the RBS of the first gene of the operon is preserved in all EGeRS1 sequences (shown in dark red, and situated in the right part of the image). Except for EGeRS1-A, the other EGeRS1 regions are predicted to form a second upstream branch of about 200 nt containing two ramifications (shown at the left part of the image in light red). The 131 bp duplication present in EGeRS1-C affects the secondary structure of this region (shown in black and without base pairs). The 5′ and 3′ ends are indicated; the RBS signal is located just upstream of the 3′ end of the alignments.

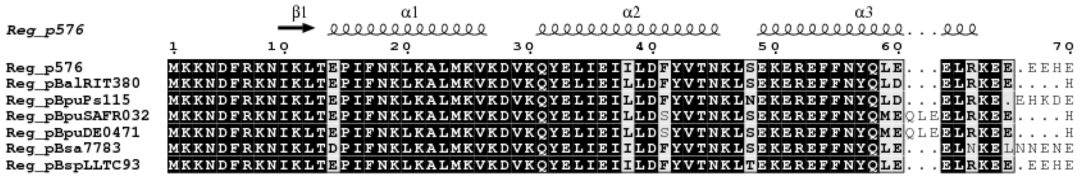

Supplemental Figure S4. All seven clade II pLS20 family plasmids encode a homolog of Reg_{p576} that in p576 is responsible for regulating the establishment genes. An alignment is shown for the Reg homologs encoded by the clade II pLS20 family plasmids. Residues conserved in all seven proteins are shown in a black background. Residues forming β-sheet and the three α-helices in Regp576 are shown at the top.

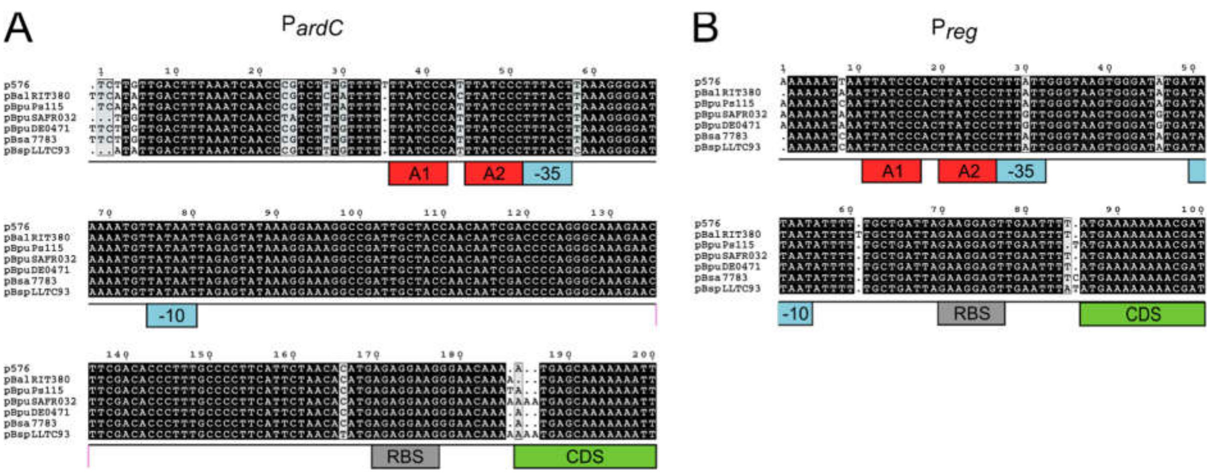

Supplemental Figure S5. Conserved promoters flanked by the Reg operators in the seven clade II plasmids of the pLS20 family. Multiple alignments of the sequences located upstream of the *ardC* (A) and the *reg* genes (B). Base pairs conserved in all seven plasmids are shown in a black background. The Reg_{p576} operator sequence of p576 is characterized by a dual copy of the 5′-TTATCCC-3′ heptamer sequence separated by two bps. The heptamers are indicated with red boxes labelled A1 and A2. The -35 and -10 boxes of the promoters as determined for plasmid p576 are indicated with blue boxes labelled -35 and -10, respectively. Ribosomal binding sites are indicated with grey rectangles labelled RBS. The 5′ regions of the downstream located *ardC* (A) and *reg* (B) genes are indicated with green rectangles labelled CDS.

Supplemental Table S1. Strains used

bacterium	strain	genotype	reference
E. coli	XL1-Blue	*endA1 gyrA96(nalR) thi-1 recA1 relA1 lac glnV44 F'[::Tn10 proAB+ lacIq Δ(lacZ)M15] hsdR17(rK- mK+)*	Bullock WO, Fernández JM and Short JM (1987)
B. subtilis	PKS11	*trpC2*, plasmid pLS20cat	Singh PK et al (2012)
	AND101	*trpC2, amyE*::P*spank* -sfGFP (spec R)	Miguel-arribas et al (2021)
	JV62A	*trpC2, amyE*::P*spank* - EGeRS1(B)$_{830A}$ -*sfGFP* (*spec*^{R})	This work
	JV62B	*trpC2, amyE*::P*spank* - EGeRS1(B)$_{830B}$ -*sfGFP* (*spec*^{R})	This work
	JV63A	*trpC2, amyE*::P*spank* - EGeRS1(B)$_{648A}$ -*sfGFP* (*spec*^{R})	This work
	JV63B	*trpC2, amyE*::P*spank* - EGeRS1(B)$_{648B}$ -*sfGFP* (*spec*^{R})	This work
	JV64A	*trpC2, amyE*::P*spank* - EGeRS1(B)$_{406A}$ -*sfGFP* (*spec*^{R})	This work
	JV64B	*trpC2, amyE*::P*spank* - EGeRS1(B)$_{406B}$ -*sfGFP* (*spec*^{R})	This work
	JV65A	*trpC2, amyE*::P*spank* - EGeRS1(B)$_{215A}$ -*sfGFP* (*spec*^{R})	This work
	JV65B	*trpC2, amyE*::P*spank* - EGeRS1(B)$_{215B}$ -*sfGFP* (*spec*^{R})	This work
	JV66	*trpC2, amyE*::P*spank* - EGeRS1(B)$_{209}$ -*sfGFP* (*spec*^{R})	This work
	JV67	*trpC2, amyE*::P*spank* - EGeRS1(B)$_{318}$ -*sfGFP* (*spec*^{R})	This work
	JV68	*trpC2, amyE*::P*spank* - EGeRS1(B)$_{542}$ -*sfGFP* (*spec*^{R})	This work
	JV69	*trpC2, amyE*::P*spank* - EGeRS1(B)$_{723}$ -*sfGFP* (*spec*^{R})	This work

Supplemental Table S2. Plasmids

Name	Description	Reference
pLS20*cat*	Native plasmid pLS20 labelled with Cm resistance cassette in unique *Sal*I site	Itaya et al., 2006
pAND101	pDR110 derivative containing *sfGFP* reporter gene cloned into *Nhe*I and *Spe*I sites	Miguel-arribas et al (2021)
pJV70	pAND101 derivative containing intergenic region *82*c-*85* (946 bp) cloned in *Sal*I site. The fragment was cloned maintaining the natural orientation of the Clue-B region towards the *sfGFP* reporter gene	This work
pJV62A	pAND101 derivative containing EGeRS1(B) region (830 bp) cloned in *Sal*I site. The fragment was cloned maintaining the natural orientation of the EGeRS1(B) region towards the *sfGFP* reporter gene	This work
pJV62B	pAND101 derivative containing EGeRS1(B) region (830 bp) cloned in *Sal*I site. The fragment was cloned with the opposite orientation of the EGeRS1(B) region towards the *sfGFP* reporter gene	This work
pJV63A	pAND101 derivative containing EGeRS1(B) region with a 5′-end deletion (648 bp) cloned in *Sal*I site. The fragment was cloned maintaining the natural orientation of the EGeRS1(B) region towards the *sfGFP* reporter gene	This work
pJV63B	pAND101 derivative containing EGeRS1(B) region with a 5′-end deletion (648 bp) cloned in *Sal*I site. The fragment was cloned with the opposite orientation of the EGeRS1(B) region towards the *sfGFP* reporter gene	This work
pJV64A	pAND101 derivative containing EGeRS1(B) region with a 5′-end deletion (406 bp) cloned in *Sal*I site. The fragment was cloned maintaining the natural orientation of the EGeRS1(B) region towards the *sfGFP* reporter gene	This work
pJV64B	pAND101 derivative containing EGeRS1(B) region with a 5′-end deletion (406 bp) cloned in *Sal*I site. The fragment was cloned with the opposite orientation of the EGeRS1(B) region towards the *sfGFP* reporter gene	This work
pJV65A	pAND101 derivative containing EGeRS1(B) region with a 5′-end deletion (215 bp) cloned in *Sal*I site. The fragment was cloned maintaining the natural orientation of the EGeRS1(B) region towards the *sfGFP* reporter gene	This work
pJV65B	pAND101 derivative containing EGeRS1(B) region with a 5′-end deletion (215 bp) cloned in *Sal*I site. The fragment was cloned with the opposite orientation of the EGeRS1(B) region towards the *sfGFP* reporter gene	This work
pJV66	pAND101 derivative containing EGeRS1(B) region with a 3′-end deletion (209 bp) cloned in *Sal*I site. The fragment was cloned maintaining the natural orientation of the EGeRS1(B) region towards the *sfGFP* reporter gene	This work
pJV67	pAND101 derivative containing EGeRS1(B) region with a 3′-end deletion (318 bp) cloned in *Sal*I site. The fragment was cloned maintaining the natural orientation of the EGeRS1(B) region towards the *sfGFP* reporter gene	This work
pJV68	pAND101 derivative containing EGeRS1(B) region with a 3′-end deletion (542 bp) cloned in *Sal*I site. The fragment was cloned maintaining the natural orientation of the EGeRS1(B) region towards the *sfGFP* reporter gene	This work
pJV69	pAND101 derivative containing EGeRS1(B) region with a 3′-end deletion (723 bp) cloned in *Sal*I site. The fragment was cloned maintaining the natural orientation of the EGeRS1(B) region towards the *sfGFP* reporter gene	This work

Supplemental Table S3. Oligonucleotides used

Name	Sequence 5′→ 3′ *	Purpose
pDR111_U	TGACTTTATCTACAAGGTGTGGC	Forward primer to amplify insert inside *Sal*I site from vector pAND101 and sequencing
opKSsfGFP	TTCCGGCATGGCGGACTTGAAGAAGTC	Reverse primer to amplify insert inside *Sal*I site from vector pAND101 and sequencing
oJV110	cgcgGTCGACCTCTCTTACATGTTCACTAGATAATACTCG	Forward primer to amplify region EGeRS1(B) and insert it into pAND101 SalI site. Use with reverse primer oJV114 to obtain a product size 850 bp (830 after digestion and insertion into pAND101)
oJV111	cgcgGTCGACGTTGCGTATCTACTTCTAAAAATGG	Forward primer to amplify a fragment of region EGeRS1(B) with a 5′-end deletion and insert it into pAND101 SalI site. Use with reverse primer oJV114 to obtain a product size 668 bp (648 after digestion and insertion into pAND101)
oJV112	cgcgGTCGACCAGAAAGGCTCCCGTAAAGCACCGG	Forward primer to amplify a fragment of region EGeRS1(B) with a 5′-end deletion and insert it into pAND101 SalI site. Use with reverse primer oJV114 to obtain a product size 426 bp (406 after digestion and insertion into pAND101)
oJV113	cgcgGTCGACGCCCGCCGCGTTACTATCCG	Forward primer to amplify a fragment of region EGeRS1(B) with a 5′-end deletion and insert it into pAND101 SalI site. Use with reverse primer oJV114 to obtain a product size 235 bp (215 after digestion and insertion into pAND101)
oJV114	cgcgGTCGACTCCTTTTTTTAAGACTTTTTTGTTTTTGCGGAGGCCGAATTGC	Reverse primer to amplify a fragment of region EGeRS1(B) and insert it into pAND101 SalI site. Use with forward primers oJV110, oJV111, JV112 and pJV113 to obtain a product sizes 850, 668, 426 and 235, respectively
oJV117	cgcgGTCGACTCCCTTACTGATTTATTCCATTCCG	Forward primer to amplify a fragment of region EGeRS1(B) and insert it into pAND101 SalI site. Use with reverse primers oJV118, oJV119, oJV120 and pJV121 to obtain a product sizes 229, 668, 426 and 235, respectively
oJV118	cgcgGTCGACATCGCTTCGCAGTTAATAATGAGGG	Reverse primer to amplify a fragment of region EGeRS1(B) with a 3′-end deletion and insert it into pAND101 SalI site. Use with forward primers oJV117 to obtain a product sizes 229 bp (209 after digestion and insertion into pAND101)
oJV119	cgcgGTCGACTTTAGAAGTAGATACGCAACTTATAGTAACTG	Reverse primer to amplify a fragment of region EGeRS1(B) with a 3′-end deletion and insert it into pAND101 SalI site. Use with forward primers oJV117 to obtain a product sizes 338 bp (318 after digestion and insertion into pAND101)
oJV120	cgcgGTCGACTGCTTCTTTTGATCTTCCGTCCTCG	Reverse primer to amplify a fragment of region EGeRS1(B) with a 3′-end deletion and insert it into pAND101 SalI site. Use with forward primers oJV117 to obtain a product sizes 562 bp (542 after digestion and insertion into pAND101)

oJV121	cgcggtcgacCGTGTTGCTTGCCGATGTTG AATGTG	Reverse primer to amplify a fragment of region EGeRS1(B) with a 3´-end deletion and insert it into pAND101 SalI site. Use with forward primers oJV117 to obtain a product sizes 743 bp (723 after digestion and insertion into pAND101)

* 5´overhang sequences are indicated in lowercase, restriction sites are underlined.

Supplemental Table S4. Putative establishment genes identified on clade II pLS20 family plasmids.

Plasmid	Protein	Size (aa)	Domains/Homology
p576	$ArdC_{p576}$	279	Antirestriction, ArdC (IPR017113)
	Reg_{p576}	70	Repressor Reg_{p576}
	p26c	68	-
	p23c	281	-
	p20c	88	-
	p19c	80	-
	p18c	88	-
pBalRIT380‡	KLV14966.1	279	Antirestriction, ArdC (IPR017113)<br>Homolog of $ArdC_{p576}$
	KLV14965.1	70	Homolog of repressor Reg_{p576}
	KLV14963.1	260	-
	KLV14961.1	88	Homolog of p20c p576 protein
	KLV14960.1	80	Homolog of p19c p576 protein
	KLV14959.1	88	Homolog of p18c p576 protein
pBpuPs115‡	RST63783.1	278	Antirestriction, ArdC (IPR017113)<br>Homolog of $ArdC_{p576}$
	RST63782.1	70	Homolog of repressor Reg_{p576}
	RST63396.1	70	-
pBpuSAFR032‡	WP_144492987.1	279	Antirestriction, ArdC (IPR017113)<br>Homolog of $ArdC_{p576}$
	WP_144492985.1	70	Homolog of repressor Reg_{p576}
	WP_144492984.1	208	-
	WP_144492981.1	280	Homolog of p23c p576 protein
pBpuDE0471†	FSY29_RS19605	111	Antirestriction, ArdC (IPR017113) [partial sequence]<br>Homolog of $ArdC_{p576}$
	WP_144492985.1	70	Homolog of repressor Reg_{p576}
	WP_144500379.1	278	Homolog of p23c p576 protein
	WP_144500381.1	88	Homolog of p20c p576 protein
	WP_144500382.1	80	Homolog of p19c p576 protein
	WP_144500383.1	88	Homolog of p18c p576 protein
pBsa7783‡	PAK32436.1	279	Antirestriction, ArdC (IPR017113)

			Homolog of ArdC$_{p576}$
	PAK32435.1	71	Homolog of repressor Reg$_{p576}$
	PAK32438.1	77	-
	PAK32413.1	279	Homolog of p23c p576 protein
	PAK32415.1	88	Homolog of p20c p576 protein
pBspLLTC93‡	PRO39502.1	279	Antirestriction, ArdC (IPR017113) Homolog of ArdC$_{p576}$
	PRO39503.1	70	Homolog of repressor Reg$_{p576}$
	PRO39506.1	280	Homolog of p23c p576 protein

Supplemental Table S5. Putative establishment genes identified on plasmids pLS20, pBatNRS213, pBamB1895, pBglSRCM103574, pBliYNP2†, pBspNMCC4† and pBS72.

Plasmid	EGeRS1	Operon position	Protein	Size (aa)	Protein family or Domains
pLS20	A	1A	79c	97	-
		2A	78c	175	SNase-like (IPR035437)
		3A	77c	265	-
		4A	76c	155	-
	B	1B	82c	280	Antirestriction, ArdC (IPR017113)
		2B	81c	85	-
	C	1C	90c	62	-
		2C	89c	85	-
		3C	88c	283	-
		4C	87c	43	-
		5C	86c	64	-
	D	1D	6c	52	-
		2D	5c	162	-
		3D	4c	80	-
		4D	3c	166	-
		5D	2c	83	-
	E	1E	9c	150	-
		2E	8c	96	-
		3E	7c	84	-
pBatNRS213	A	1A	UFD97640.1	97	-
		2A	UFD97639.1	175	SNase-like (IPR035437)
		3A	UFD97638.1	262	-
		4A	UFD97637.1	159	-
		5A	UFD97636.1	89	-
	B	1B	UFD97644.1	85	-
		2B	UFD97643.1	283	-
		3B	UFD97642.1	65	-

	C	1C	UFD97648.1	269	-
		2C	UFD97647.1	71	-
		3C	UFD97646.1	1011	N-6 adenine-specific DNA methylase (IPR003356)<br>P-loop NTPase (IPR000605)
	D	1D	UFD97654.1	52	-
		2D	UFD97653.1	222	-
		3D	UFD97652.1	80	-
		4D	UFD97651.1	166	-
		5D	UFD97650.1	85	-
		6D	UFD97649.1	87	-
	E	1E	UFD97657.1	150	-
		2E	UFD97656.1	96	-
		3E	UFD97655.1	84	-
pBamB1895	A	1A	UFD97724.1	97	-
		2A	UFD97723.1	177	SNase-like (IPR035437)
		3A	UFD97722.1	264	-
		4A	UFD97721.1	155	-
	B	1B	UFD97726.1	280	Antirestriction, ArdC (IPR017113)
		2B	UFD97725.1	85	-
	C	1C	UFD97727.1	119	-
	D	1D	UFD97733.1	51	-
		2D	UFD97731.1	63	-
		3D	UFD97730.1	87	-
		4D	UFD97729.1	93	-
		5D	UFD97728.1	87	-
	E	1E	UFD97736.1	144	-
		2E	UFD97735.1	101	-
		3E	UFD97734.1	65	-
		4E	UFD97733.1	79	-
pBglSRCM103574	A	1A	WP_128748439.1	72	-
		2A	WP_128748441.1	62	-
		3A	WP_128748443.1	110	-
		4A	WP_128748445.1	95	Winged helix DNA-binding (IPR036390)
		5A	WP_128748447.1	178	-
		6A	WP_128748449.1	178	-
	B	1B	WP_128748433.1	138	-
		2B	WP_128748435.1	245	DUF2786 (IPR024498)
		3B	WP_128748437.1	116	-
	C	1C	WP_128748555.1	262	-
		2C	WP_128748429.1	1009	N-6 adenine-specific DNA methylase (IPR003356)<br>P-loop NTPase (IPR000605)
	D	1D	WP_128748543.1	271	-
		2D	WP_128748545.1	67	-
		3D	WP_128748547.1	113	-
		4D	WP_128748549.1	85	-
	E	1E	WP_128748541.1	521	C-5 cytosine Mtase (IPR001525)
pBliYNP2†	A	1A	OJT66889.1	95	-

		2A	OJT66888.1	191	SNase-like (IPR035437)
		3A	OJT66887.1	243	-
		4A	OJT66886.1	177	-
	B	1B	OJT66896.1	72	-
		2B	OJT66895.1	64	-
		3B	OJT66894.1	110	-
	C	1C	OJT66897.1	278	Antirestriction, ArdC (IPR017113)
	D	1D	OJT66901.1	268	-
		2D	OJT66900.1	245	DUF2786 (IPR024498)
		3D	OJT66899.1	120	-
	E	1E	OJT66830.1	66	-
		2E	not annotated	114	-
		3E	OJT66829.1	88	-
	F	1F	OJT66831.1	522	C-5 cytosine Mtase (IPR001525)
pBspNMCC4†	A	1A	PRS35764.1	509	C-5 cytosine Mtase (IPR001525)
	B	1B	PRS35760.1	207	-
		2B	PRS35761.1	244	DUF2786 (IPR024498)
		3B	PRS35762.1	139	-
		4B	PRS35763.1	135	-
	C	1C	PRS35755.1	182	-
		2C	PRS35756.1	183	-
		3C	PRS35757.1	127	-
		4C	PRS35758.1	129	-
		1D	PRS35754.1	127	-
		2D	PRS35767.1	91	LPD11 (IPR040789)
	E	1E	PRS35752.1	264	-
		2E	PRS35753.1	1007	N-6 adenine-specific DNA methylase (IPR003356)<br>P-loop NTPase (IPR000605)
	F	1F	PRS35750.1	478	-
pBS72	A	1A	APB62340.1	233	-
		2A	APB62339.1	100	-
		3A	APB62338.1	156	-
		4A	APB62337.1	89	-
	B	1B	APB62343.1	63	-
		2B	APB62342.1	267	-
		3B	APB62341.1	1011	N-6 adenine-specific DNA methylase (IPR003356)<br>P-loop NTPase (IPR000605)
	C	1C	APB62349.1	86	-
		2C	APB62348.1	115	-
		3C	APB62347.1	84	-
		4C	APB62346.1	118	-
	D	1D	APB62357.1	85	-
		2D	APB62356.1	52	-
		3D	APB62355.1	224	-
		4D	APB62354.1	73	-
		5D	APB62353.1	521	C-5 cytosine Mtase (IPR001525)
